# REDUCE (Reviewing long-term antidepressant use by careful monitoring in everyday practice) internet and telephone support to people coming off long-term antidepressants: protocol for a randomised controlled trial

**DOI:** 10.1186/s13063-020-04338-7

**Published:** 2020-05-24

**Authors:** Tony Kendrick, Adam W. A. Geraghty, Hannah Bowers, Beth Stuart, Geraldine Leydon, Carl May, Guiqing Yao, Wendy O’Brien, Marta Glowacka, Simone Holley, Samantha Williams, Shihua Zhu, Rachel Dewar-Haggart, Bryan Palmer, Margaret Bell, Sue Collinson, Imogen Fry, Glyn Lewis, Gareth Griffiths, Simon Gilbody, Joanna Moncrieff, Michael Moore, Una Macleod, Paul Little, Christopher Dowrick

**Affiliations:** 1 Primary Care, Population Sciences, and Medical Education, University of Southampton, Aldermoor Health Centre, Southampton, UK; 2 Department of Health Services Research and Policy, Faculty of Public Health and Policy, London School of Hygiene and Tropical Medicine, London, UK; 3 Department of Health Sciences, University of Leicester, George Davies Centre, Leicester, UK; 4 Department for Rehabilitation and Sport Sciences, Faculty of Health and Social Sciences, Bournemouth University, Bournemouth, UK; 5 School of Psychology, Building 44 Highfield Campus, University of Southampton, Southampton, UK; 6 Division of Psychiatry, Faculty of Brain Sciences, University College London, London, UK; 7 Southampton Clinical Trials Unit, University of Southampton and University Hospitals Southampton NHS Foundation Trust, Southampton General Hospital, Southampton, UK; 8 Department of Health Sciences, Seebohm Rowntree Building, University of York, York, UK; 9 Hull York Medical School, Allam Medical Building, University of Hull, Hull, UK; 10 Institute of Psychology Health and Society, University of Liverpool, Liverpool, UK

**Keywords:** Depression, Antidepressants, Discontinuation, Withdrawal, Deprescribing, Primary care, Digital intervention

## Abstract

**Background:**

Around one in ten adults take antidepressants for depression in England, and their long-term use is increasing. Some need them to prevent relapse, but 30–50% could possibly stop them without relapsing and avoid adverse effects and complications of long-term use. However, stopping is not always easy due to withdrawal symptoms and a fear of relapse of depression. When general practitioners review patients on long-term antidepressants and recommend to those who are suitable to stop the medication, only 6–8% are able to stop. The Reviewing long-term antidepressant use by careful monitoring in everyday practice (REDUCE) research programme aims to identify safe and cost-effective ways of helping patients taking long-term antidepressants taper off treatment when appropriate.

**Methods:**

Design: REDUCE is a two-arm, 1:1 parallel group randomised controlled trial, with randomisation clustered by participating family practices. Setting: England and north Wales. Population: patients taking antidepressants for longer than 1 year for a first episode of depression or longer than 2 years for repeated episodes of depression who are no longer depressed and want to try to taper off their antidepressant use. Intervention: provision of ‘ADvisor’ internet programmes to general practitioners or nurse practitioners and to patients designed to support antidepressant withdrawal, plus three patient telephone calls from a psychological wellbeing practitioner. The control arm receives usual care. Blinding of patients, practitioners and researchers is not possible in an open pragmatic trial, but statistical and health economic data analysts will remain blind to allocation. Outcome measures: the primary outcome is self-reported nine-item Patient Health Questionnaire at 6 months for depressive symptoms. Secondary outcomes: depressive symptoms at other follow-up time points, anxiety, discontinuation of antidepressants, social functioning, wellbeing, enablement, quality of life, satisfaction, and use of health services for costs. Sample size: 402 patients (201 intervention and 201 controls) from 134 general practices recruited over 15–18 months, and followed-up at 3, 6, 9 and 12 months. A qualitative process evaluation will be conducted through interviews with 15–20 patients and 15–20 practitioners in each arm to explore why the interventions were effective or not, depending on the results.

**Discussion:**

Helping patients reduce and stop antidepressants is often challenging for practitioners and time-consuming for very busy primary care practices. If REDUCE provides evidence showing that access to internet and telephone support enables more patients to stop treatment without increasing depression we will try to implement the intervention throughout the National Health Service, publishing practical guidance for professionals and advice for patients to follow, publicised through patient support groups.

**Trial registration:**

ISRCTN:12417565. Registered on 7 October 2019.

**Administrative information**

**Table:** 

Title {1}	REDUCE (reviewing long-term antidepressant use by careful monitoring in everyday practice): protocol for a randomised controlled trial
Trial registration {2a and 2b}.	Title: Trial of internet and telephone support to people coming off long-term antidepressants. Trial ID: ISRCTN12417565 Date registered: 7 October 2019. Link: www.isrctn.com/ISRCTN12417565
Protocol version {3}	Version 1.4 dated 28 November 2019
Funding {4}	National Institute for Health Research (NIHR) Programme Grant for Applied Research (PGfAR; ref. RP-PG-1214-20,004)
Author details {5a}	Tony Kendrick, Professor of Primary Care, ark1@soton.ac.uk; Adam WA Geraghty, Senior Research Fellow, A.W.Geraghty@soton.ac.uk; Hannah Bowers, Research Fellow, H.M.Bowers@soton.ac.uk; Beth Stuart, Associate Professor, bls1@soton.ac.uk; Geraldine Leydon, Professor of Medical Sociology, G.M.Leydon@soton.ac.uk; Wendy O’Brien, Programme Manager, W.Obrien@soton.ac.uk; Samantha Williams, Senior Research Assistant, S.J.Williams@soton.ac.uk; Shihua Zhu, Senior Research Fellow in Health Economics, S.Zhu@soton.ac.uk; Rachel Dewar-Haggart, Senior Research Assistant, R.V.Dewar-Haggart@soton.ac.uk; Brian Palmer, patient and public involvement (PPI) representative, bryan@palmeradaptation.co.uk; Margaret Bell, PPI representative, embellmrs@btinternet.com; Sue Collinson, PPI representative, sue.collinson@homerton.nhs.uk; Michael Moore, Professor of Primary Health Care Research, M.V.Moore@soton.ac.uk; Paul Little, Professor of Primary Care Research, p.little@soton.ac.uk **Primary Care, Population Sciences, and Medical Education, University of Southampton, Aldermoor Health Centre, Southampton SO16 5ST, UK** Carl May, Professor of Medical Sociology, carl.may@lshtm.ac.uk **Department of Health Services Research and Policy, Faculty of Public Health and Policy, London School of Hygiene and Tropical Medicine, 15–17 Tavistock Place, London WC1H 9SH, UK** Guiqing Yao, Professor of Health Economics, gy38@leicester.ac.uk **Department of Health Sciences, University of Leicester, George Davies Centre, University Road, Leicester LE1 7RH, UK** Marta Glowacka, Lecturer in Occupational Therapy and Health Psychology, mglowacka@bournemouth.ac.uk; **Department for Rehabilitation and Sport Sciences, Faculty of Health and Social Sciences, Royal London House R601, Christchurch Road, Bournemouth BH1 3LT, UK** Simone Holley, Research fellow, S.L.Holley@soton.ac.uk; Imogen Fry, Undergraduate Honorary Assistant Psychologist, ijf1g17@soton.ac.uk **School of Psychology, Building 44 Highfield Campus, University of Southampton, Southampton SO17 1BJ, UK** Glyn Lewis, Professor of Epidemiological Psychiatry, glyn.lewis@ucl.ac.uk; Joanna Moncrieff, Professor of Critical and Social Psychiatry, j.moncrieff@ucl.ac.uk **Division of Psychiatry, Faculty of Brain Sciences, University College London, 6th Floor, Maple House, 149 Tottenham Court Rd, London W1T 7NF, UK** Gareth Griffiths, Professor of Clinical Trials, G.O.Griffiths@soton.ac.uk **Southampton Clinical Trials Unit, University of Southampton and University Hospitals Southampton NHS Foundation Trust, Southampton General Hospital, Tremona Road, Southampton SO16 6YD, UK** Simon Gilbody, Professor of Psychological Medicine and Health Services Research, Simon.Gilbody@york.ac.uk **Department of Health Sciences, Seebohm Rowntree Building, University of York, Heslington, York, YO10 5DD, UK** Una Macleod, Dean and Professor of Primary Care Medicine, Una.Macleod@hyms.ac.uk **Hull York Medical School, Allam Medical Building, University of Hull, Hull HU6 7RX, UK** Christopher Dowrick, Professor of Primary Medical Care, cfd@liverpool.ac.uk **Institute of Psychology Health and Society, University of Liverpool, Liverpool L69 3GL, UK**
Name and contact information for the trial sponsor {5b}	Dr Alison Knight, Head of Research Governance, Research Governance Office, University of Southampton, Room 4079, Building 37, Highfield Campus, Southampton SO17 1BJ, UK. Tel: 0238059 5058. Fax: 0238059 5781. Email: rgoinfo@soton.ac.uk
Role of sponsor {5c}	The study sponsor (the University of Southampton) and funder (NIHR) were not involved in the study design, writing of the protocol paper, or the decision to submit for publication.

## Introduction

### Background and rationale {6a}

Antidepressant prescriptions have risen steadily year on year since 1990 because general practitioners (GPs) have been prescribing longer and longer courses [1, 2], and the average length of treatment is now more than 2 years. Some people need long-term antidepressants to prevent relapse, but surveys suggest 30–50% have no evidence-based indication for long-term use [3]. However, stopping is not easy due to withdrawal symptoms including anxiety and mood changes, which feel similar to the reason why treatment was started in the first place [4]. Patients on long-term treatment are often given repeat prescriptions and are reviewed only infrequently [5, 6].

Taking antidepressants over the long term exposes patients to the risks of side effects. Common side effects of antidepressants include changes in weight, changes in sleep and sexual dysfunction [7]. Less commonly, some patients develop bleeding from the stomach or intestine, and the use of antidepressants in people aged over 65 years is associated with an increase in the risks of falls, seizures and strokes [8]. Around one in two patients on antidepressants feel emotional blunting or numbness [9]; thus, the drugs should not be continued over the long term unless there is a good reason for taking them.

Antidepressants constitute a substantial proportion of the National Health Service (NHS) drug budget (2.5% in 2010 [10]), and the costs of unnecessary treatment include appointments for medical or nursing reviews. The cost of GP care for depression was estimated to be £200 m per year in 2006 [11], in addition to the cost of the antidepressant prescriptions of around £300 m per year, so substantial savings could be made if significant numbers of long-term users were to discontinue.

Many patients are dissatisfied with this situation and would like help to stop long-term treatment. However, GPs often lack experience in reducing antidepressant medication flexibly, and GP advice to taper and stop treatment is not often successful. Prompting GPs to review patients eligible for stopping treatment was tested in a trial in the Netherlands and found to be ineffective, with only 6% of patients discontinuing in the intervention group and 8% in the control group [12, 13]. Similarly, an uncontrolled trial of pharmacist-prompted GP review of long-term users in Scotland resulted in only 7% stopping [14]. Therefore, it appears that without a specific intervention addressing issues with patient and practitioner behaviours, many patients will continue antidepressants unnecessarily. Practitioners need guidance to provide support for tapering and stopping treatment, and patients need further support and advice on coping with withdrawal symptoms.

The aim of the REDUCE programme is to identify feasible, safe, effective and cost-effective ways of helping patients taking long-term antidepressants taper and stop treatment when appropriate.

### Objectives {7}

1. To determine the effectiveness of the intervention through a randomised controlled trial
2. To estimate cost-effectiveness from a health and personal social service perspective, with a sensitivity analysis from a societal perspective.

### Trial design {8}

REDUCE is a two-arm, 1:1 parallel group randomised controlled trial, with randomisation clustered by participating family practices to avoid contamination between intervention and control arms.

## Methods: participants, interventions and outcomes

This is protocol version 1.4, dated 28 November 2019.

### Study setting {9}

The study setting is primary care (group general family practices) in England and north Wales recruited from the Universities of Southampton, Liverpool and Hull. A full list of study sites can be obtained by email from reduce@soton.ac.uk.

### Eligibility criteria {10}

Our aim is to include patients who are taking long-term antidepressant treatment that is not indicated according to the National Institute for Health and Care Excellence depression guideline [15]. We will therefore include all consenting patients on treatment for more than 1 year for a first episode, and patients treated for more than 2 years for a recurrent episode, who are no longer depressed or judged to be at significant risk of relapse.

The significant risk factors for relapse are: current significant depressive symptoms on the nine-item Patient Health Questionnaire (PHQ-9; see below) despite antidepressant treatment, current significant anxiety symptoms on the seven-item Generalised Anxiety Disorder (GAD-7) rating scale (see below), current suicidal ideas (see below) and current psychiatric outpatient or inpatient treatment for depression.

In addition to the above criteria increasing the risk of relapse, the following are also exclusion criteria: bipolar disorder, comorbid psychosis, substance use, dementia as a primary diagnosis, spoken or written English language inadequate to take part in interviews or complete questionnaires, and another indication for taking antidepressants (e.g. neuropathic pain).

### Who will take informed consent? {26a}

Patients on long-term antidepressants are identified through searches of computerised practice medical record databases, mailed a patient information sheet (PIS) about the study by the practice and asked to contact the study team if they wish to take part, or to decline, using a reply slip and a freepost envelope after they have had time to consider. If they do not respond the research team will have no knowledge of them, maintaining patient confidentiality, and this will not prejudice their future treatment. If patients do respond positively to either approach, a member of the research team will then contact them, screen them by telephone for any exclusion criteria, and arrange to see them face to face for a baseline visit if they are eligible. At baseline, the researcher will go over the PIS again and seek formal written consent.

All patients are told we are recruiting people who have been taking antidepressants for more than 1 year for a first episode, or more than 2 years for a recurrent episode, with a view to working out how to help them reduce their medication, if appropriate, with the advice of their practice GP or nurse. The PIS outlines the two different approaches in the intervention and control arms, but not in detail, and potential participants do not know at this point to which arm their general practice has been randomised. This is to avoid differential rates of consent to the two arms based on patients’ opinions of the intervention or procedures involved in each.

Having consented to take part in writing on this initial basis, and having undergone baseline assessment, patients will then be given further information about the details of the procedures in the arm to which their practice has been randomised.

Patients who do not wish to consent to take part at that point for reasons that may be temporary (i.e. they do wish to try to reduce and stop taking their antidepressant, but not at that time due to current life stresses, recent life events, or the timing of upcoming events and so forth) have the option of consenting to be re-contacted after 3 months to be asked again if they wish to participate at that later point.

### Additional consent provisions for collection and use of participant data and biological specimens {26b}

This trial does not involve collecting biological specimens for storage.

### Interventions

#### Explanation for the choice of comparators {6b}

The intervention consists of GP or nurse practitioner (NP) and patient access to ‘ADvisor’ internet programmes designed to support antidepressant withdrawal, plus three patient telephone calls from a psychological wellbeing practitioner. Control patients receive usual care without internet or telephone support, but patients are prompted to seek a review of their long-term antidepressant treatment at the start of their involvement in the trial and may also decide to try to taper off their antidepressant use in discussion with their GP or NP.

#### Intervention description {11a}

The practitioner intervention (called ‘ADvisor for Health Professionals’ as it gives advice about antidepressants) includes internet modules on: why reduce; broaching the subject; when to start tapering; reduction schedules for individual antidepressants; dealing with withdrawal symptoms; dealing with relapse; a summary of the ADvisor intervention for patients; and printable pages on antidepressant reduction regimes and sections of ADvisor for patients that are recommended for the patients to consult. The intervention broadly targets increasing the self-efficacy of GPs and NPs to safely discontinue patients from antidepressants where appropriate. It was developed using evidence and theory in combination with in-depth qualitative interviews/focus groups with GPs/NPs. A paper describing the development of the health professional intervention is being prepared for submisison for publication in another journal, which is what we mean by 'elsewhere'.

The patient intervention (called ADvisor) has also been developed drawing on theory, evidence and in-depth systematic qualitative research with patients, and has been more fully described elsewhere [16]. As in the health professional intervention, our aim is to increase patients’ self-efficacy for stopping antidepressants in a way that is safe and suited to their preferences. We focused on increasing patients’ reflective motivation for stopping, and supporting their psychological and physical capability to do so through internet modules that include: reducing and stopping (introduction to website); how to reduce antidepressants; thinking about antidepressants (their effects and why lifelong treatment may not be necessary); dealing with withdrawal symptoms; I am worried about stopping; keeping well; thinking about what you value in life; and moving forward.

In the intervention arm practices, the GPs/NPs are given access and an introduction to the online ADvisor for Health Professionals and induction to the study, which is either practice-based or online. Through reading ADvisor for Health Professionals, they receive education on best practice in the supervision of antidepressant tapering and cessation, focussing on the differences between withdrawal symptoms and relapse, and the management of withdrawal symptoms.

As patients are recruited to the intervention arm they are asked to make an appointment with their GP/NP to discuss coming off their antidepressant and agreeing an initial dosage tapering schedule. The number and timing of subsequent GP/NP consultations during tapering and following drug cessation is left to the participating GPs/NPs to agree with the patients on an individual basis.

In addition to the ADvisor internet modules and GP/NP consultations, the following telephone support is provided to patients in the intervention arm by a trained psychological wellbeing practitioner:

- Call 1 (0–2 weeks), for 30 min: to check the patient’s understanding of the ADvisor intervention and encourage confidence in going through the tapering and cessation process
- Call 2 (4–6 weeks), for 15 min: to ask the patient how tapering is going and whether they are following the schedule and, where necessary, to advise the patient to discuss any issues with tapering with their GP
- Call 3 (timing agreed with patient), for 15 min: to ask the patient about any residual withdrawal symptoms and go over techniques to help with relapse prevention.

A sample of 10–20 telephone calls per practitioner will be audiorecorded in the first 3 months, and again halfway through the trial, to check for fidelity against the telephone support guide.

In the control arm, participating practices are informed that the recruited patients are potentially eligible for tapering off antidepressants. Their electronic medical records are flagged and, as they are recruited, patients are asked to make an appointment as part of usual care to see their GPs/NPs for a review of the need for their antidepressant medication, but the practitioners are not given the ADvisor for Health Professionals information on best practice in tapering, unlike practitioners in the intervention arm.

Alerting the control arm practices to the potential eligibility of patients for tapering off antidepressants will result in some patients tapering and ceasing treatment. This will be permitted within the trial. Our power calculation for the secondary outcome of antidepressant discontinuation in the main trial assumes a 7% discontinuation rate in the control practices, which is the rate found in previous studies of simply prompting GPs to review patients potentially eligible for discontinuation [13, 14].

#### Criteria for discontinuing or modifying allocated interventions {11b}

There are no specific criteria for discontinuing or modifying allocated interventions at the time of writing. The Independent Data Monitoring Committee (IDMC) will monitor the progress of the trial, decide whether any interim analyses are necessary, decide whether there are circumstances under which the trial should be stopped, and make their recommendations to the Programme Steering Committee (PSC).

#### Strategies to improve adherence to interventions {11c}

The three psychological wellbeing practitioner telephone calls are aimed at encouraging patients in the intervention arm to use the internet support. Patients’ use of the internet programme is automatically recorded to allow estimation of adherence to the intervention.

#### Relevant concomitant care permitted or prohibited during the trial {11d}

Practitioners are free to refer patients to other sources of help in both arms (e.g. referral for psychological therapy).

#### Provisions for post-trial care {30}

Participating patients remain under the usual care of the treating practitioner.

### Outcomes {12}

Outcome measures are collected at baseline and at 3, 6, 9 and 12 months (see Table 1 for exact timings). The primary outcome is the PHQ-9 score for depressive symptoms at 6 months. We will also consider this outcome using a repeated measures approach over the full 12 months of follow-up with a generalised linear mixed model allowing for responses clustered within participants over time and participants clustered within practices. Secondary outcomes are: anxiety; discontinuation of antidepressants (at 6 months, for at least 2 months); mental wellbeing; antidepressant withdrawal symptoms; antidepressant side effects; patient satisfaction; patient enablement; quality of life; and use of health services to calculate costs.

**Table 1.** Data collection summary

Measure	Baseline (face to face)	3 months (postal or online)	6 months (face to face)	9 months (postal or online)	12 months (face to face)
Sociodemographics and past history of depression questionnaire	√				
Depression (PHQ-9)	√	√	√	√	√
Anxiety (GAD-7)	√	√	√	√	√
Suicidal ideas	√	√	√	√	√
Discontinuation of antidepressants (for at least 2 months, by 6 months)			√		
Quality of life (EQ-5D-5L, SF-12)	√	√	√	√	√
Wellbeing (WEMWBS)	√		√		√
Withdrawal symptoms (DESS)	√	√	√		
Antidepressant side effects (ASEC, CSFQ-C) (if taken)	√		√		√
Satisfaction (MISS-29)			√		√
Enablement (PEI)			√		√
Questionnaires on use of services, use of antidepressants and sickness absence	√		√		√
Beliefs about antidepressants questionnaire	√	√			√
Collective efficacy questionnaire		√			

*ASEC* antidepressant side-effects check-list, *CSFQ-C* Changes in Sexual Functioning Questionnaire, *DESS* Discontinuation Emergent Signs and Symptoms, *EQ-5D-5L* EuroQol five dimensions with five levels, *GAD-7* seven-item Generalised Anxiety Disorder, *MISS-29* 29-item Medical Informant Satisfaction Scale, *PEI* Patient Enablement Instrument, *PHQ-9* nine-item Patient Health Questionnaire, *SF-12* 12-item Short Form, *WEMWBS* Warwick–Edinburgh Mental Wellbeing Scale

The PHQ-9 [17] is a self-complete questionnaire taking approximately 3 min to complete. It measures nine core symptoms of depression based on the Diagnostic and Statistical Manual fourth edition criteria and has high sensitivity and specificity in UK primary care [18].

The GAD-7 is a brief instrument for measuring anxiety, which has also been validated in primary care [19]. Although originally developed to detect GAD, it also has good sensitivity and specificity for panic, social anxiety, and post-traumatic stress disorders [20].

Discontinuation of antidepressants is deemed to have occurred once the patient has stopped taking them for 2 months. Determining discontinuation after 6 months allows tapering and cessation to take up to 4 months. In our experience, after 2 months, withdrawal symptoms will have mostly gone, and mood problems will have re-emerged if they are going to. We believe patients who decide to resume treatment are unlikely to wait even 1 month.

We are using the Warwick–Edinburgh Mental Wellbeing Scale (WEMWBS) [21] as an additional secondary outcome measure since this measures both subjective experiences of happiness and life satisfaction (the ‘hedonic perspective’), and positive psychological functioning, good relationships with others and self-realisation (the ‘eudaimonic perspective’). The latter includes capacity for self-development, positive relations with others, autonomy, self-acceptance and competence. Coming off long-term antidepressants might improve these aspects of wellbeing since side effects can include emotional blunting ,which should be reduced, and a greater sense of autonomy and self-acceptance might result from not having to rely on medication. WEMWBS has been shown to be responsive to change with a range of mental health interventions in specialist and community populations [22].

Antidepressant withdrawal symptoms are measured using the Discontinuation Emergent Signs and Symptoms scale, a brief self-report measure on which participants can indicate the presence of, and changes in, 43 possible antidepressant withdrawal symptoms [23]. Withdrawal symptoms are measured (for all patients, whether or not they withdraw from antidepressants) at 3 and 6 months, asking patients to rate their presence looking back over the period since recruitment.

Antidepressant side effects are measured at baseline and at 6 and 12 months (for all patients, whether or not they withdraw from antidepressants) using the antidepressant side-effects check-list developed by Aitchison as part of the GENDEP research project (http://gendep.iop.kcl.ac.uk/results.php). It asks participants to rate the presence of 21 possible side effects and also includes open questions for other symptoms not listed, and demonstrates good agreement between self-report and psychiatrists’ ratings [24]. At baseline and at 6 and 12 months we also use the Changes in Sexual Functioning Questionnaire, a 14-item self-rating instrument including five domains of sexual functioning, which has been shown to be reliable and valid in both clinical and research settings [25, 26].

Patient satisfaction is assessed using the 29-item Medical Interview Satisfaction Scale which was developed in the USA to assess patient satisfaction with individual doctor–patient consultations and has been shown to be valid and reliable in UK primary care [27]. We have adapted it to rate patient satisfaction at the 6-month follow-up, asking patients to look back over their consultations with the GPs/NPs for advising on tapering and cessation of antidepressants.

We use the Patient Enablement Instrument that is designed to capture patients’ ability to understand the nature of their problems and cope with their illness [28]. This was developed in primary care to be completed by the patient after a consultation. We have adapted it to rate patient enablement at the 6-month follow-up, looking back over the whole period of the intervention, as was done successfully in the ATEAM trial [29] when the adapted measure proved sensitive to change.

We are measuring quality of life using the EuroQol five dimensions with five levels (EQ-5D-5L) measure [30]. The EQ-5D-5L is the measure favoured by the National Institute for Health and Care Excellence in determining cost effectiveness when developing its clinical guidelines. The EQ-5D-5L includes five dimensions: mobility, self-care, usual activities, pain/discomfort, and anxiety/depression, each scored on five levels (no problems, slight problems, some problems, severe problems and extreme problems). It is an improvement on the original three-level EQ-5D-3L which was developed to reduce ceiling effects experienced with the EQ-5D-3L. We will apply the EQ-5D-5L scoring algorithm and value set for England to translate EQ-5D-5L data scores into utility scores [31].

We also assess quality of life using the Medical Outcomes Study-derived measure of functional health status, the 12-item Short Form [32], from which utility scores can be derived using the six-item Short Form [33]. One reason for using both measures is that the six-item Short Form may be more sensitive to changes in quality of life related to mild depression than the EQ-5D-5L [34]. We also do not know which measure will be more sensitive to changes in quality of life resulting from relief from antidepressant side effects on the one hand, or withdrawal symptoms on the other, resulting from antidepressant discontinuation. We therefore use the 12-item Short Form in addition to the EQ-5D-5L in the trial and explore the implication of quality of life gained or lost in our study population in a sensitivity analysis.

The health economics analysis is being undertaken from an NHS and personal social services perspective, with a sensitivity analysis from a societal perspective including time lost from work. Bespoke questionnaires are used to collect data on health and social service resource use, personal out of pocket spending, and time off work. In addition, a review of patients’ computerised GP records is conducted by practice staff at the end of the patient’s involvement in the study to extract any additional health service usage including medication, primary care consultations, outpatient appointments, accident and emergency department attendances, and hospital admissions. All items will be costed using appropriate data (e.g. British National Formulary, Personal Social Service Research Unit and NHS reference costs), with informal care costed at minimum wage level. The resource-use questionnaire is administered face to face with patients at baseline and at 6 and 12 months, asking them to look back over the previous 6 months at each point. The case note review is done by general practice staff at the end of the 12 months of follow-up.

#### Mediator and moderator measures

In addition to the outcomes listed above, a bespoke questionnaire asking for patients’ beliefs about antidepressants, and their cessation, developed by a Southampton PhD student (RD-H), is administered at the face-to-face assessments at baseline and at 3 and 12 months. This will enable a mediator analysis of possible effects of changes in patients’ beliefs on changes in antidepressant use.

At the 3-month assessment point, we also administer the collective efficacy questionnaire [35] which is a measure of the strength of support for discontinuing antidepressants that a participant perceives among their important friends and family, and a possible moderator of the success of the intervention.

### Participant timeline {13}

**Fig. 1.**
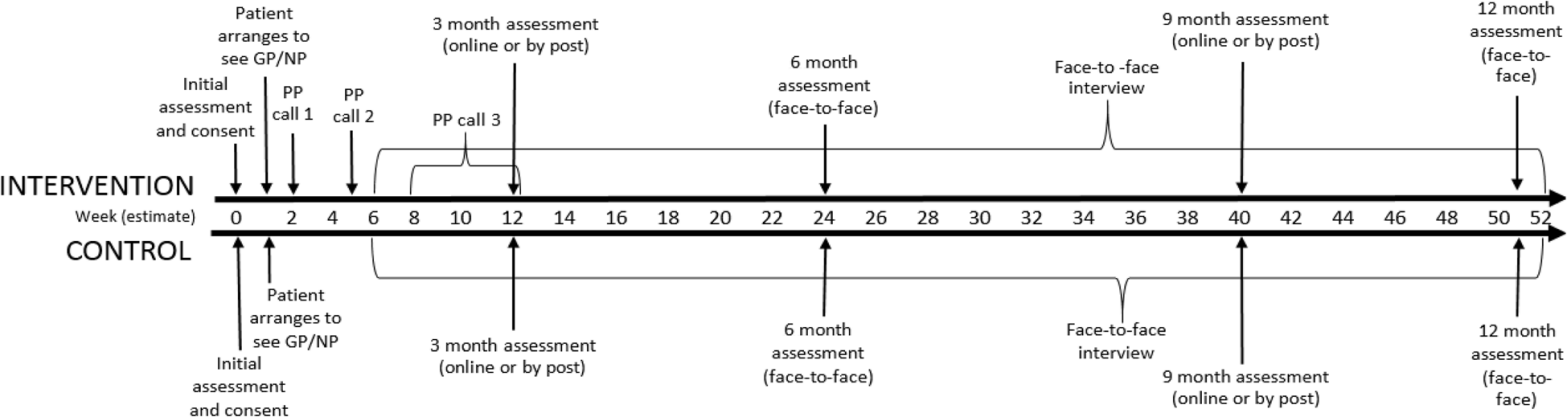
The participant timeline for the study. GP general practitioner, NP nurse practitioner, PP psychological practitioner

The participant timeline is shown in Fig. 1.

### Sample size {14}

To have 90% power, with a one-sided alpha of 2.5%, to establish non-inferiority in terms of depressive symptoms within two points (estimated to be the minimal clinically important difference) on the PHQ-9 at 6 months (standard deviation 5.4), we need 155 patients followed up in each arm. Assuming a variable cluster size of between 1 and 7 per practice (mean 3) and an intra-cluster correlation coefficient of 0.012 (from the Health Technology Assessment THREAD trial of treating mild-to-moderate depression in primary care [36]), gives a 1.033 cluster design effect (based on a coefficient of variation of 1.5/3 = 0.5, using the formula of Eldridge [37]). Anticipating 20% do not comply with the intervention and/or are lost to follow-up, we need to randomise (155 × 2 × 1.036) / 0.8 = 402 patients (201 per arm) from 134 practices (67 per arm).

### Recruitment {15}

Potential patient participants are approached in two ways: 1) through practice records database searches; and 2) opportunistically in GP or NP consultations.

All eligible patients identified by searches are actively approached in both intervention and control arms to avoid the risk of selection bias inherent in relying only on opportunistic recruitment by practitioners.

Practice computerised medical record databases are searched using ‘Read’ diagnostic and symptom codes for depressive diagnoses and symptoms, together with British National Formulary chapter codes for antidepressants taken over the previous 2 years. Standardised searches were developed for the two main practice computer systems SystmOne and EMIS, which can be given to participating practices. Participating GPs check the lists of potential participants against the inclusion and exclusion criteria to ensure all are suitable to be invited to take part.

Mail-out packs to patients include an invitation letter from the GP, the participant information leaflet (PIL) and a reply slip for the patients to complete indicating whether they are interested in taking part. We use the Docmail digital mail-out facility where possible. Interested patients are asked to contact the REDUCE team directly using the reply slips (in freepost envelopes) or by email. If patients do not contact the research team then the team has no knowledge of their names or addresses, thus maintaining patient confidentiality.

Eligible patients may also be invited to consider taking part within a GP or NP consultation for depression. Patients are provided by the practitioner with a pack including the GP invitation letter, PIL and reply slip to be returned directly to the research team using a freepost envelope if the patient is interested in discussing possible involvement in the trial. Again, there is no contact between the research team and potential patients unless the patients initiate it.

Those patients who return reply slips to the research team indicating a willingness to discuss possible participation are contacted by a member of the research team by telephone and screened for the exclusion criteria. This involves asking a standard set of yes/no questions and administration of the PHQ-9 for depressive symptoms and the GAD-7 for anxiety symptoms (see below). Patients are reminded of the information provided to them in the PIL sent with the GP invitation letter, and if they have no exclusion criteria they are asked for a convenient time to meet with a member of the research team.

We ask the participating general practice if they would like to send a reminder invitation to participate letter to participants who do not respond to the first invitation to participate. We think that some participants may not be ready to taper their antidepressants when they receive the first invitation for reasons that may be transient, for example too close to Christmas. As people’s situations and personal circumstances fluctuate, a follow-up letter may be received at a time that the participant considers more appropriate for tapering.

### Assignment of interventions: allocation

#### Sequence generation {16a}

Randomisation of practices will be by computerised sequence generation, and minimisation with a random element using three factors to avoid imbalance between the two arms: practice size (large/small), location (urban/rural) and social deprivation (dichotomised around the median Index of Multiple Deprivation score). The allocation ratio is 1:1 but there is a random element to the minimisation algorithm and so we might not expect perfect balance to the randomisation.

#### Concealment mechanism {16b}

The allocation sequence is generated by the Southampton Clinical Trials Unit separate to the research teams recruiting the practices.

#### Implementation {16c}

Notification of allocation is online by the Clinical Trials Unit. The research teams enrol the participant practices, and the Clinical Trials Unit assigns participant practices to intervention or control arms.

### Assignment of interventions: blinding

#### Who will be blinded {17a}

Blinding of both patients and practitioners in the intervention arm during patient recruitment and face-to-face assessment is impossible given the cluster randomisation of practices and the nature of the intervention. Self-report outcome measures are therefore being used to prevent observer rating bias by research team members aware of the patient’s assigned trial arm. The statisticians and health economists analysing the data are kept blind to allocation.

Telephone follow-up where necessary will be carried out by a research assistant in a different university to the recruiting university, blind to practice allocation, who will advise the patients on first contact not to reveal which arm of the trial they are in. Any inadvertent unblinding will be recorded and reported. The trial research assistants will also obtain information from medical records, but at the end of the study in order not to unblind them to practice allocation during patient follow-up.

#### Procedure for unblinding if needed {17b}

The statisticians and health economists analysing the data will be unblinded to allocation only after all the data have been collected, entered into the database, and cleaned.

### Data collection and management

#### Plans for assessment and collection of outcomes {18a}

Patients are initially screened by telephone and excluded if they have any of the exclusion criteria. These include significant depressive symptoms despite antidepressant treatment defined as a score of 12 or more on the PHQ-9 completed over the telephone. Patients are also excluded if they have significant anxiety at baseline, i.e. a score of 10 or more on the GAD-7, also completed over the telephone. If patients score above 0 (i.e. 1, 2 or 3) on the ninth question of the PHQ-9 about suicide/self-harm they are also excluded from participating, and this information is relayed to their GP immediately for them to discuss this, preferably with their permission. The information may be relayed without their permission if necessary, after discussion between the researcher, the principal investigator and the patient.

The other exclusion criteria addressed through telephone screening of patients include: current psychiatric outpatient or inpatient treatment for depression (yes/no); comorbid psychosis, substance use or dementia as a primary diagnosis (yes/no); spoken or written English language inadequate to take part in interviews or complete questionnaires (yes/no); and another indication for taking antidepressants, e.g. neuropathic pain (yes/no).

Data collection at baseline assessment is face to face but data are entered online using i-survey (University of Southampton secure online survey system) where possible. Data collection at 3 and 9 months are through either online i-survey or postal follow-up, with one reminder after 2 weeks, and subsequent face-to-face or telephone follow-up to obtain the outcomes for patients who do not complete the i-survey or return their questionnaires by post. Postal questionnaires are accompanied by an explanatory letter requesting return of completed questionnaires within 2 weeks if possible. Data collection at 6 and 12 months is face to face. Participants are given a £10 gift voucher for their time at the first (face-to-face) assessment and again at the final 12 -month (face-to-face) follow-up. Relevant information on consultations at the practice and use of services outside the practice is also extracted from patients’ medical records by practice staff at the end of the study, assuming the patients have given consent for this.

#### Data collection summary

The data collection summary is shown in Table 1.

#### Plans to promote participant retention and complete follow-up {18b}

We ask participants when we screen them if they are happy to receive texts. If participants are happy to disclose their mobile number, we keep in regular contact with them using the University of Southampton text messaging service. Text messages are sent to remind participants about appointments and completing online questionnaires.

If patients are uncontactable when trying to arrange their face-to-face follow-ups at 6 and 12 months, postal questionnaires are sent together with a shopping voucher, and a compliment slip offering a telephone call to complete the main outcome questionnaires (PHQ-9 and EQ-5D-5L) if that is preferable to the patient. Patients’ practices are also contacted with a request to practice staff to help the research team contact patients who are uncontactable. If the patient has not been contactable within 6 weeks of a follow-up point, they are deemed to have been lost to follow-up at that point, but the same rigorous approaches are made again at the next follow-up point.

#### Data management {19}

Participant data are entered on laptop computers on site and then transferred to electronic databases and stored at the University of Southampton. Data stored are checked for missing or unusual values (range checks) and checked for consistency within participants over time. Any suspect data are returned to the researcher or practice in the form of data queries.

#### Confidentiality {27}

Participant data are pseudo-anonymised by assigning each participant an identifier code used to identify the participant during the study and for any participant-specific clarification between the University of Southampton as sponsor and participating general practices.

The informed consent form specifies the participant data to be collected and how it is managed or might be shared, including handling of all patient identifiable data and sensitive patient identifiable data adhering to relevant data protection law. Only trained personnel assigned specific roles are granted access to the electronic patient data.

Data will be retained at the University of Southampton in accordance with the General Data Protection Regulation (2018) act. The participants’ medical records and other relevant data may also be reviewed by appropriate qualified personnel independent from the trial team who are appointed to audit the study, including representatives of the competent authority. Details will remain confidential and participants’ names will not be recorded outside the university.

#### Plans for collection, laboratory evaluation and storage of biological specimens for genetic or molecular analysis in this trial or for future use {33}

This trial does not involve collecting biological specimens for storage.

### Statistical methods

#### Statistical methods for primary and secondary outcomes {20a}

A full and detailed statistical analysis plan will be developed prior to the final analysis of the trial. The main features of the statistical analysis plan are as follows.

Intention-to-treat (ITT) analyses at the patient level will be performed using mixed logistic/linear regression models, controlling for baseline values, stratification variables and potential confounders as appropriate. Practices will be modelled as a random effect to allow for the clustering of patients within practices. Patterns of missing data will be explored, sensitivity analysis will be used to explore the impact of missingness, and imputation of missing data will be used. In a non-inferiority trial where some patients do not comply with treatment as randomised, the difference between the arms can appear reduced and the groups look more similar, leading to the incorrect conclusion of non-inferiority. A per-protocol analysis would analyse individuals based on their compliance with treatment as randomised, excluding non-compliant participants, giving a more conservative estimate of effect for non-inferiority [38]. However, the exclusion of some participants after randomisation can potentially lead to bias. Therefore, we will present both intention-to-treat and per-protocol analyses.

We will also undertake a complier-average causal effect analysis, which is another approach for dealing with non-compliance that compares compliant participants in the intervention group with those in the control group whose characteristics are similar enough to the intervention group compliers to suggest they too would have complied with the intervention given the opportunity to do so [39]. Compliance for these analyses in the intervention arm will be defined as completing the first session of the LifeGuide programme within 6 months of recruitment (anticipating >90%). The first session will have information about antidepressant treatment, the rationale for attempting withdrawal, and how withdrawal should be attempted under supervision. We would expect patients to benefit from that session even if they do not log on again. Compliance in the control arm will be defined as having consulted the GP/NP to have their antidepressant treatment reviewed within the 6-month follow-up period.

For the primary outcome, we will report the analyses based on all three approaches and interpret the findings cautiously in light of any differences between approaches that may emerge. This analysis will form the core of the publication of the trial results.

For secondary outcomes, the analyses will use a similar modelling approach to that set out for the primary outcome, with mixed logistic/linear regression models as appropriate, with a random effect for practice, controlling for baseline values, stratification variables and potential confounders as appropriate. Discontinuation of antidepressants will be evaluated at the 6-month time point. All other secondary measures will be analysed using a repeated measures approach with measures clustered within patients over time in addition to the clustering of patients within practice. The models will control for stratification variables and potential confounders as appropriate and, where a baseline measure for the outcome is available, will control for the baseline value as well.

#### Health economics analysis

For the full trial, our proposed economic evaluation will be taken from an NHS and personal social services perspective with a sensitivity analysis from a societal perspective. The outcome will be expressed as incremental cost per point improvement in the PHQ-9 clinical outcome, incremental cost per discontinuation of antidepressants, and incremental cost per quality-adjusted life year (QALY) gained (cost utility analysis). Case note review at 12 months will augment the 6-monthly patient reports of health and social service resource use. All items will be costed using appropriate data (e.g. Personal Social Service Research Unit, NHS reference costs), with informal care costed at minimum wage level. The primary analysis will be at 12 months. Personal costs will include patient and carer time off work, personal expenses, use of the internet, and travel. Itemised resource usage will be weighted by associated unit costs and aggregated over 12 months. QALYs will be estimated by the area under the curve approach.

A generalised linear mix model will be used to estimate the differences in costs and QALYs (using both EQ-5D-5L and six-item Short Form utilities), adjusting for baseline characteristics including socio-economic deprivation and internet use. Where appropriate, we will estimate incremental cost-effectiveness ratios. We will estimate mean values and 95% percentiles using non-parametric bootstrapping, and use these to estimate cost-effectiveness acceptability curves. Major assumptions in the costing and quality of life analysis will be tested through sensitivity analyses. A decision analytic model will be developed to extrapolate the cost effectiveness beyond the trial period covering the potential risk of recurrence if the intervention proves to be effective in terms of improvements in QALYs up to 12 months. The health economics analysis will be published.

#### Interim analyses {21b}

No a priori interim analyses are planned. Full details of the analyses to be undertaken will be set out in a statistical analysis plan, to be approved by the independent PSC. The IDMC will review outcome and safety data regularly during the trial, advising the PSC on continuation of the trial.

#### Methods for additional analyses (e.g. subgroup analyses) {20b}

No a priori subgroup analyses are planned. Any post-hoc analyses will be exploratory only.

A qualitative process evaluation will also be carried out. Process evaluation is an important tool for understanding both the dynamics and the outcomes of clinical trials, and the normalisation process theory (NPT) [40] is a conceptual toolkit developed for this purpose. NPT focuses on understanding the mechanisms that promote, and the factors that inhibit, sense-making, participation, action and monitoring by participants in implementation processes.

The objectives of the process evaluation in the trial are to identify, characterise, and explain the perspectives of patient and practitioner participants on the conduct of the trial, and to construct a taxonomy of factors affecting both the conduct of the trial and the potential for normalisation of the use of the interventions in everyday practice, outside of the trial situation. The analysis will enable the construction of an implementation framework of barriers and facilitators (patient and health system factors) that need to be taken into account in the use of the interventions in primary care practice.

The qualitative interviews will be transcribed and emerging themes identified through inductive analysis using the constant comparative method. We will draw on insights from the wide range of studies that have employed NPT, giving a basic structure to the topic guide to be written in advance of the interviews. However, we will also work prospectively and inductively to ensure that we identify, characterise and understand 1) disconfirming evidence and deviant cases, and 2) processes that are not accounted for within NPT.

#### Methods in analysis to handle protocol non-adherence and any statistical methods to handle missing data {20c}

We will examine the structure and pattern of missing data and, if appropriate, will present a sensitivity analysis based on data imputed using a multiple imputation model. As described above, data will be analysed on an intention-to-treat basis, on a per-protocol basis, and using a complier-average causal effect analysis.

#### Plans to give access to the full protocol, participant-level data and statistical code {31c}

The anonymised quantitative datasets (but not the qualitative interview data) generated during the current study may be available upon request from TK (ark1@soton.ac.uk) from 31 August 2023, depending on the types of analyses planned and submission of a peer-reviewed, funded, and ethically approved proposal. The trial dissemination group, whose purpose is to oversee the planned outputs from the trial and agree on whether data are shared, comprises the Chief Investigator (TK) in Southampton and one co-applicant from each of the other two centres: Liverpool (CD) and Hull (UM).

### Oversight and monitoring

#### Composition of the coordinating centre and trial steering committee {5d}

TK, CD and UM lead regular local study team meetings at Southampton, Liverpool and Hull, respectively, and overall REDUCE Management Team Meetings take place every month through teleconferencing to review progress and give advice on the conduct and management of the study. The Management Team Meeting includes representatives with expertise in general practice, psychiatry, psychology, sociology, statistics and health economics, and is supported by three PPI contributors and Clinical Trials Unit staff involved in the day-to-day running of the trial.

An independent PSC has been set up to oversee trial conduct, consisting of an academic GP (chair), academic psychologist, statistician and two patient representatives.

#### Composition of the data monitoring committee, its role and reporting structure {21a}

The IDMC consists of an academic psychiatrist (chair), academic GP and a statistician. The IDMC will review outcome and safety data regularly during the trial, advising the PSC on continuation of the trial.

#### Adverse event reporting and harms {22}

Any adverse events reported by patients or practitioners will be brought to the attention of the Trial Coordinator and Chief Investigator (CI) or, in the absence of the CI, one of the Principal Investigators (PIs). The CI or PI will decide whether or not to inform sponsor or the Research Ethics Committee (REC), PSC or IDMC. The report will include the event, when the information was reported, assessment of seriousness and likely relationship to participation in the trial.

All serious adverse events will be reported to the CI and the Trial Coordinator within 24 h of the local site becoming aware of the event. We will record the nature of the event, date of onset, severity, corrective therapies given, outcome, causality (i.e. unrelated, unlikely, possible, probably, definitely) and expectedness. The CI will assign the causality and expectedness of the event and the term should be in accordance with the latest version of MedDRA and grades given in accordance with the NCI CTCAE v4.03. Additional information will be provided as soon as possible if the event has not resolved at the time of reporting.

The CI or Programme Manager will notify the REC of related and unexpected serious adverse events occurring during the study according to the following timelines: fatal and life-threatening within 7 days of notification and non-life threatening within 15 days. Adverse events will also be reported to the IDMC who will advise the PSC about continuation and whether interim analyses are needed. The PSC will work with the IDMC and be kept informed by the CI, PI, or Trial Coordinator. If an extension was requested it would be the responsibility of the PSC to look in detail as to why this was needed and give an opinion which would inform the funder (NIHR) and the sponsor (University of Southampton).

#### Frequency and plans for auditing trial conduct {23}

The trial teams at Southampton, Liverpool and Hull undertake a number of internal audits of their own systems and processes regularly. In addition, participants’ trial records, medical records and other relevant data may be reviewed by appropriate qualified personnel independent from the trial teams appointed to audit the study, including representatives of the sponsor and of the Health Research Authority. Details will remain confidential and participants’ names will not be recorded outside the universities.

#### Plans for communicating important protocol amendments to relevant parties (e.g. trial participants, ethical committees) {25}

Proposed important protocol modifications (e.g. changes to eligibility criteria, outcomes and analyses) will be discussed with the co-investigators before seeking approval from the Health Research Authority and REC, and subsequently communicated to the trial registry and journals in any publications arising.

### Dissemination plans {31a}

The results of the REDUCE trial will be disseminated to participating practices in summary form, as well as academic audiences via publication in peer-reviewed journals and general practice trade publications. We will also publicise our findings through existing primary care networks and patient groups. Summary trial results will be available on the websites of the participating universities. The trial dissemination group, whose purpose is to oversee the planned outputs from the trial and agree on whether data are shared, comprises the CI TK in Southampton and one co-applicant from each of the other two centres: Liverpool (CD) and Hull (UM).

## Discussion

Helping patients reduce and stop antidepressants is often challenging for practitioners and time consuming for very busy primary care practices. If providing internet and telephone support enables more patients to stop treatment without increasing depression we will spread the intervention throughout the NHS, publishing practical guidance for professionals and advice for patients to follow, publicised through patient support groups.

### Trial status

This paper is based on version 1.4 of the protocol, dated 28 November 2019, when the REDUCE trial is still recruiting. The protocol was initially approved by the North of Scotland Research Ethics Committee (REC 1) on 2 September 2019 (reference no. 19/NS/0144). A substantial amendment was approved by the REC and Health Research Authority on 16 December 2019. Recruitment of practices began on 1 December 2019, and the approximate date when recruitment of patients will be completed is 30 June 2021. The end of the study is defined as the date of the last follow-up visit of the last patient (expected to occur 12 months after the last patient is recruited).

## Abbreviations

CI: Chief Investigator
EQ-5D-5L: EuroQol five dimensions with five levels
GAD-7: Seven-item Generalised Anxiety Disorder
GP: General practitioner
IDMC: Independent Data Monitoring Committee
NHS: National Health Service
NP: Nurse practitioner
NPT: Normalisation process theory
QALY: Quality-adjusted life year
PHQ-9: Nine-item Patient Health Questionnaire
PI: Principal Investigator
PIL: Patient information leaflet
PIS: Patient information sheet
PPI: Patient and public involvement
PSC: Programme Steering Committee
REC: Research Ethics Committee
REDUCE: Reviewing long-term antidepressant use by careful monitoring in everyday practice
WEMWBS: Warwick–Edinburgh Mental Wellbeing Scale
